# A Case of Upper Limb Osteomyelitis and Septic Arthritis Presenting as Pseudoparalysis in a Two-Week-Old

**DOI:** 10.1155/2018/1796831

**Published:** 2018-11-12

**Authors:** Kuldeep Stohr, Guang Xia Xu

**Affiliations:** Trauma and Orthopaedics Department, Cambridge University Hospitals Trust, Cambridge, UK

## Abstract

Pseudoparalysis presenting in infants is a rare manifestation, where infection and trauma are the principle differentials. We present a case of a two-week-old baby boy with pseudoparalysis who was initially diagnosed as Erb's palsy when presented in the emergency department and later re-presented with signs of sepsis. A later diagnosis of osteomyelitis of the humerus and septic arthritis of the shoulder was made. Despite antibiotic therapy and surgical drainage, the proximal epiphysis of his humerus remains abnormal; however, he has no apparent functional deficit of his right arm at four-year follow-up.

## 1. Introduction and Case

We present a case of a two-week-old baby boy who presented to an emergency department with decreased movements of his right arm. This lack of spontaneous movement of the right upper limb had occurred within the last 36 hours. He had been delivered vaginally, and there were was no report of a shoulder dystocia or an instrumented delivery. At the time of presentation, he was apyrexic and feeding normally and he showed no overt signs of systemic illness. Blood tests were performed, and the only abnormal finding was an elevated CRP of 132 mg/L (normal 0–6 mg/L). A diagnosis of Erb's palsy was made, and the patient was discharged home with an outpatient physiotherapy referral.

**Figure 1:**
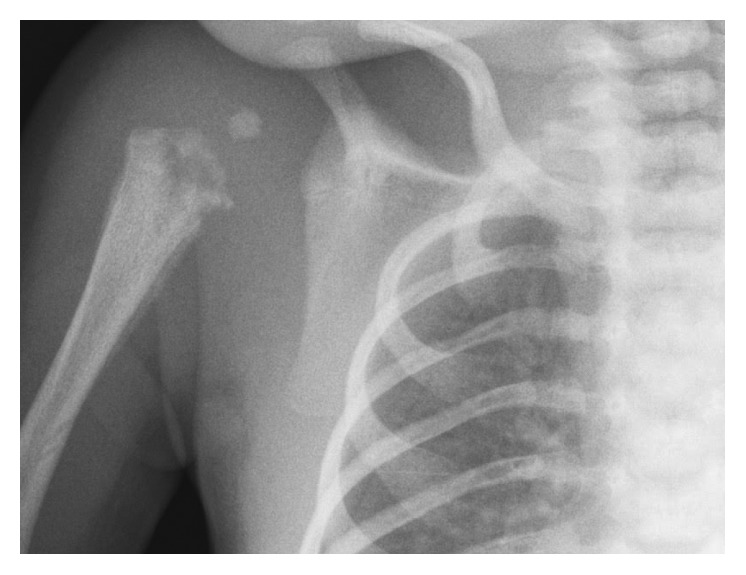
X-ray imaging of right shoulder at second presentation.

He presented five days later; this time, he was unwell with clear signs of sepsis. X-rays (Figure 1) and MRI scan showed profuse osteomyelitis of the humerus with septic arthritis of the shoulder. *E*. *coli* was cultured. He received surgical drainage and six weeks of intravenous antibiotics. He regained right-arm movements two days after surgery. The proximal epiphysis of his humerus remains abnormal; however, his upper limb lengths and movements are symmetrical at recent four-year follow-up.

## 2. Discussion

Pseudoparalysis presenting in infants is a concerning symptom, where infection and trauma are the chief differentials. Clinical suspicion is especially crucial as infants do not necessarily present with fever or the traditional signs of sepsis. Furthermore, laboratory results may only be slightly deranged, although in this infants' case a CRP of 132 should have raised initial suspicion or at least have prompted further investigation or repeat of the result. Birth trauma, most commonly a clavicle fracture, can cause pseudoparalysis. However, this would have presented immediately at birth. In the case of this infant, we saw paralysis at the age of two weeks in a seemingly well child where the only anomaly was the elevated CRP.

It is therefore important to consider the following points: (a) whether CRP is a valid marker for the diagnosis of sepsis in neonates and (b) the presentation of Erb's palsy (a type of brachial plexus birth injury).

### 2.1. Is CRP a Valid Inflammatory Marker in Neonates?

The role of CRP as a diagnostic marker for neonatal sepsis has been widely studied and analysed. CRP is a protein produced in the liver, a process induced by IL6, or through an interaction with IL1 [1]. The rate of hepatocellular synthesis of CRP increases within hours and can reach a thousand fold of its baseline, and levels may remain high if the insult persists. In neonates, a rise in CRP is always indicative of endogenous synthesis, as its placental transmission rates are extremely low [2].

Generally, when analysing the use of a marker, it is essential to review its sensitivity and specificity values. As a marker for neonatal sepsis, a low sensitivity (i.e., not picking up cases of neonatal sepsis where it is present) is generally more detrimental than a low specificity (overtreating cases where it is not present). Unfortunately, there are vastly different reported values for CRP sensitivities and specificities, ranging from 29 to 100% and 6 to 100%, which partially limits this analysis [3, 4]. However, there is a general consensus that, although it may have a lower sensitivity in the early stage of disease due to its delayed synthesis, CRP is particularly useful for monitoring the response to treatment. CRP is therefore best used in the context of the clinical picture and any available culture results and in itself (without further investigation) should not be the only criteria to initiate antibiotic therapy [5].

In our case, the initial result of 132 mg/L should have prompted further investigations.

### 2.2. Erb's Palsy

Erb's palsy refers to a brachial plexus injury affecting the upper trunk nerves (C5 and C6). The injury itself is often as a result of shoulder dystocia and breech or difficult deliveries (although delivery was normal in the case of this infant). This manifests itself with a “waiter's tip deformity” (inability to abduct or to internally rotate the shoulder, as well as inability to supinate the forearm) at birth. The key to a diagnosis of Erb's palsy is the birth history and the fact that the signs occur at birth. There are no reported cases of a brachial plexus birth injury in a normal-sized baby with an atraumatic delivery. Isolated case reports have also detailed the occurrence of brachial plexus injury seen with septic arthritis or osteomyelitis where the nerve roots are directly involved in the infection. In the 2011 case report by Mascarenhas et al., this detailed a seven-week-old infant who presented with pseudoparalysis of his right arm. This infant was subsequently diagnosed with *Staphylococcus aureus* neonatal sepsis, osteoarticular infection complicated with brachial plexus neuropathy was considered, and MRI and electromyography confirmed the diagnosis [6].

## 3. Recommendations

We recommend that pseudoparalysis itself be taken as a more specific sign for joint or bone infections in babies and those presenting with this symptom should automatically have a full sepsis screening, including imaging. Furthermore, an isolated rise in CRP combined with pseudoparalysis is indicative of infection.

## References

[1] Weinhold B., Rüther U. (1997). Interleukin-6-dependent and -independent regulation of the human C-reactive protein gene. *Biochemical Journal*.

[2] Hofer N., Zacharias E., Müller W., Resch B. (2012). An update on the use of C-reactive protein in early-onset neonatal sepsis: current insights and new tasks. *Neonatology*.

[3] Jaye D. L., Waites K. B. (1997). Clinical applications of C-reactive protein in pediatrics. *Pediatric Infectious Disease Journal*.

[4] Hengst J. M. (2003). The role of C-reactive protein in the evaluation and management of infants with suspected sepsis. *Advances in Neonatal Care*.

[5] Philip A. G., Mills P. C. (2000). Use of C-reactive protein in minimizing antibiotic exposure: experience with infants initially admitted to a well-baby nursery. *Pediatrics*.

[6] Mascarenhas A., Almeida C., Constantino C., Soudo A. P., Calado E., Vieira J. P. (2011). Septic arthritis presenting as brachial plexus neuropathy. *BMJ Case Reports*.

